# The Forty-Sixth Annual Meeting of the Mississipi Valley Association of Dental Surgeons

**Published:** 1890-05

**Authors:** 


### PROCEEDINGS.

### The Forty-Sixth Annual Meeting of the Mississippi Valley Association of Dental Surgeons.

The association met in Lincoln Club Hall, Cincinnati, Ohio, Wednesday morning March 5th, 1890, at 10 o'clock, with the President, Dr. J. R. Callahan, of Cincinnati, O., in the chair.

Prayer was offered by Dr. Jas. Leslie, of Cincinnati.

Minutes of the last session were read and approved.

The committee on revision of the constitution of the society was called and continued.

The treasurer, Dr. Frank Hunter, reported the balance on hand at close of last meeting to be $115.40.

The president appointed Drs. E. G. Betty and L. E. Custer, a committee to audit the treasurer's account, which they subsequently reported correct and their report was accepted.

Upon motion, 2 o'clock on Thursday was set apart as the hour for the exhibition of appliances.

Vice-Pres. Dr. M. H. Fletcher taking the chair, Pres. Dr. J. R. Callahan, made the opening address. It was discussed by Drs. J. Taft, H. A. Smith, M. H. Fletcher, Jas. Leslie, H. J. McKellops, C. M. Wright and others.

On motion of Dr. J. Taft, the president with Dr. H. J. McKellops, were made a committee to report on the advisability of appointing additional members in the executive committee of the association.

Under the head of miscellaneous business, which was then taken up, Dr. J. Taft spoke upon the subject of the International Dental Congress which brought forth some discussion.

Upon motion of Dr. F. A. Hunter, the hours for the present meeting were made 9–12 A. M. 2–5 P. M. Adjourned to meet at 2 P. M. this day.

AFTERNOON SESSION.

Association met at 2 P. M., Pres. Dr. J. R. Callahan in the chair.

Minutes of the morning session were read and approved.

The president appointed Drs. J. Taft, E. G. Betty, A. F. Emminger a Committee on Necrology.

Dr. Otto Arnold, of Columbus, O., read a paper upon "Non-Metallic Plastic Materials for Filling Teeth." Discussed by Drs. J. S. Cassidy, H. A. Smith, J. Taft, C. M. Wright, H. J. McKellops, L. E. Custer, Frank Hunter, W. H. Sillito and others.

The next paper was "Inventions and New Things," by Dr. J. Taft, Cincinnati. Discussed by Drs. H. J. McKellops, H. A. Smith and others.

Then followed a paper by Dr. C. M. Wright, of Cincinnati—"The Wealth of the Dentist." Discussed by Drs. Frank Hunter, James Leslie, H. J. McKellops, H. A. Smith and Frank Sage.

Adjourned to meet at 10 o'clock on Thursday.

MORNING SESSION, THURSDAY.

Association met at 10 o'clock with the President, Dr. J. R. Callahan in the chair.

Minutes of the previous session were read and approved.

Drs. H. J. McKellops and J. R. Callahan, the committee appointed to report upon the advisability of appointing additional members upon the Executive Committee of the Association made the following suggestion :

Your committee appointed for the purpose of offering some plan to increase the attendance and interest in the society would respectfully suggest a revision of the Executive Committee as follows; that one member be appointed on the committee in each of the following cities :

Pittsburgh, Wheeling, Cleveland, Toledo, Detroit, Chicago, St. Louis, Indianapolis, Louisville, Nashville, Columbus and two in Cincinnati ; and that each of these members be expected to furnish something of interest for the meeting (either a paper or clinic) for the year in which they are appointed, and that the committee be expected to begin work early and keep the dental journals of the Mississippi Valley posted in regard to their work, and that they shall endeavor to make the next meeting of the association the largest and most profitable in its history, and that the treasurer be instructed to honor all reasonable bills of this committee to an amount not exceeding ($50) fifty dollars, (each bill), and that the committee be instructed to change the time of meeting of this association to correspond with that of the Alumni Association of the Ohio College of Dental Surgery.

Respectfully submitted,

(Signed) H. J. McKellops,
J. R. Callahan.

The paper, "Professional Ethics and Honor," by Dr. W. Storer, now of Philadelphia, was read by the secretary. It was discussed by Drs. Frank Sage, H. A. Smith, E. G. Betty, H. J. McKellops, J. Taft and Otto Arnold.

A paper, "Veterinary Dental Surgery and Operative Dentistry," by Dr. L. A. Anderson, of Cincinnati, was accompanied by an interesting clinic and followed by full discussion. On motion a vote of thanks was tendered Dr. Anderson.

Adjourned to meet at 2 P. M.

AFTERNOON SESSION CLINICS, THURSDAY.

The association met at 2 P. M. and devoted the afternoon to the consideration of instruments and appliances.

The minutes of the morning session were read and approved.

Dr. A. S. Billings, of Omaha, Nebraska, demonstrated the construction of the Chase Metallic Combination Plate, and also a ready method of obtaining a metal die.

Dr. Jas. Leslie, of Cincinnati, read a paper, "Crystalline Gold vs. Gold Foil," and showed some specimens of gum facings made by Dr. Verier, of Weymouth, England.

Dr. H. J. McKellops presented for examination a book in the German text—probably the oldest work on dentistry extant—also a set of Darby-Perry Excavators, a special set of chisels and excavators, wooden handles, various machine mallets, new separators, cervix clamps, new brush wheel, matrix holder, and the cord engine.

Dr. L. E. Custer, of Dayton, Ohio, exhibited an improved pneumatic plugger.

Dr. L. P. Bethel showed a matrix retainer made by Dr. Ivory and an improved form of the Barnes mallet.

Dr. E. G. Betty, of Cincinnati, exhibited the H. J. McKellops blotter for recording dental operations, and specimens of matrices made by Dr. Barnes, of Cleveland, O.

On motion, a vote of thanks was tendered Dr. McKellops for this exhibition of appliances.

Adjourned to meet at the dental college at 9 o'clock A. M. on Friday.

At the Friday morning session at the dental college the members witnessed a successful demonstration of a new bracket gas furnace by Dr. J. R. Callahan.

The members then adjourned to Lincoln Hall and continued the session, with President Callahan in the chair.

Minutes of previous session were read and approved.

Dr. M. H. Fletcher, of Cincinnati, read a paper on "A System of Crown and Bridge Work," following with a demonstration of the method, which was fully discussed.

On motion of Dr. E. G. Betty the following was adopted:

*Resolved:*—That the by-laws of the association be amended to the effect that fifteen dollars shall be the annual salary paid to the recording secretary.

The Committee on Necrology, Drs. J. Taft, E. G. Betty and W. H. H. Hunter, presented the following report upon the death of Dr. Wm. M. Hunter, of Cincinnati, O.; Dr. F. H. Rehwinkle, of Chillicothe, O.; Dr. A. Berry, of Cincinnati, O.; Dr. W. A. Pease, of Dayton, O.; Dr. H. L. Moore, of Cincinnati, O.; Dr. R. L. Evans, Toledo, O., and Dr. C. R. Taft, of Cincinnati, O. The report was unanimously adopted and a copy of each resolution ordered sent to the respective families of the deceased.

DR. WM. M. HUNTER, OF CINCINNATI.

Dr. Wm. M. Hunter, in whose death the society loses one of its oldest and earliest members, was one of the old-school gentlemen, whose like is rapidly vanishing from the world. He came upon the professional arena at a time when just such as he were needed to furnish brains and assistance to the young practitioner. He it was, as well as Dr. John Allen, who gave this country the perfection of methods for working "continuous gums," a process that we have since developed merely by modifying its application.

In making this resolution the association conveys to the family of the deceased the keen sense of its loss, for to us, and the profession at large, his name is a bright star in the galaxy with which the Mississippi Valley Association of Dental Surgeons has illumined the professional world.

(Signed) E. G. Betty, J. Taft, W. H. H. Hunter. } Committee.

DR. A. BERRY, OF CINCINNATI.

Dr. A. Berry, of Cincinnati, O., died in Boston, Mass., Oct. 12th, 1889. A meeting of the dentists residing in the Mississippi Valley was called, and met Aug. 13th, 1844, for the purpose of organizing the Mississippi Valley Association of Dental Surgeons. One of the members present at this preliminary meeting was Dr. A. Berry, then of Raymond, Miss., and he was from that day until his death an active member of the association, and very rarely, if ever, absent from its meetings. There never was of the hundreds who had membership in the Association any one with greater devotion to its interests than Dr. Berry. He was not only an active member of this society but he was an interested participant in association work, when it came within his reach, and not only was he a devotee to this, but he was interested in every thing that pertained to the art and science of dentistry. He was a man of culture and refinement, and was a frequent contributor to the literature of the profession.

In the Death of Dr. Berry this society has lost a valuable, honorable and enthusiastic member. The place of such a man can not easily be filled. We desire in this manner to give expression to our high regard and esteem entertained for Dr. Berry. We would furthermore express our sincerest sympathy with the friends and family of our beloved brother.

*Resolved*, That this record be sent, in proper form by the secretary, to the family of the deceased.

(Signed) J. TAFT, E. G. BETTY, A. F. EMMINGER. } Committee.

DR. F. H. REHWINKLE, CHILLICOTHE, O.

WHEREAS, it has pleased the Allwise Dispenser of all things to remove from us our esteemed friend and brother, Dr. F. H. Rehwinkle, we can not do less than give expression to the high regard entertained for him as a member of this body.

He was tender and gentle as a child, yet bold, strong and vigorous in the maintenance of the right. He possessed a moral nature of a high order; he occupied an enviable position as a public spirited citizen, always having in his charge more or less of the responsibility of public affairs, and this not only of his own city and vicinity, but of the State and country as well. In all his relations of life, public, professional, social and domestic, he was one to be emulated by all. In the possession of this combination of qualities there is, perhaps, no where to be found his superior, and but seldom his equal.

Therefore, *Resolved*, That we can not do less than to thus give expression and put upon record the evidence of our sorrow, and sense of loss, in the removal of him who was one of the oldest, most active and efficient members of this Society.

Further *Resolved*, That we most heartily commend his qualities, aspirations and activities of life as well worthy of emulation by all.

We recommend that this preamble and resolution be engrossed in a memorial page of the book of proceedings of this society, also, that a copy, properly prepared, be sent to the family of our departed brother.

(Signed) J. TAFT, C. R. BUTLER, H. A. SMITH. } Committee.

DR. W. A. PEASE, DAYTON, O.

Our brother and co-laborer, W. A. Pease, M D., D.D.S., who departed this life at Dayton, Ohio, was a graduate of the Boston

College of Medicine, and was for some time a practitioner of medicine. He afterward adopted the dental profession and always maintained, sought and upheld a high standard of ethics, and a courteous affiliation with those who held high views of the dental profession. Up to the time of his death he was a constant student and a contributor to dental literature. He was always ready and willing to be an instructor to those who sought his aid.

He practiced dentistry in Dayton, Ohio, since 1846, up to the time of his death. At one time he accumulated a fortune of $100,000, which was afterwards lost in speculation, so that he left only a small estate for his two sons. We, the members of the Mississippi Valley Association of Dental Surgeons, tender to his family, relatives and friends, our heartfelt sympathy in their loss.

(Signed) W. H. H. HUNTER,<br>J. TAFT,<br>E. G. BETTY. } Committee.

DR. CALVIN R. TAFT, OF CINCINNATI, O.

Since the last meeting of this society, Almighty God in His infinite wisdom has seen fit to call hence our companion, friend and professional brother, Dr. Calvin R Taft. Dr. Taft was in many respects a superior man, conscientious, earnest and faithful to a fault in all he undertook, and ever watchful of the interests of the profession he loved. To know him was to love and respect him for his many virtues. He was a true friend to all who tried to elevate his profession, and possessed that courage of his conviction to such a degree that he was always ready to battle for what he thought to be right. The best years of his life were given early in the strife, for the defense of the flag he loved so well, and but few knew how much he suffered in after-life from the exposure of army life, for he was not a man to complain even to his most intimate friends.

Therefore be it *Resolved*, That the sincerest sympathy of this society be extended to the family of the deceased and that this record be spread upon the minutes of the association, and a copy sent to his family.

(Signed) A. F. EMMINGER,<br>J. TAFT,<br>E. G. BETTY. } Committee.

DR. R. L. EVANS, TOLEDO, O.

Dr. R. L. Evans was one of our members, and was a man well and favorably known to the profession in this part of the country. He was a good dentist, was always welcomed at our meetings, though not taking a very active part in official work.

It is resolved that the association extend to the family our sympathy and that a copy of these resolutions be transmitted to his family.

(Signed) E. G. BETTY, J. TAFT, A. F. EMMINGER. } Committee.

DR. H. L. MOORE, OF CINCINNATI, O.

Dr. H. L. Moore was one of the younger members of the profession, one who was most earnest and conscientious in his daily work. His first studies in dentistry were undertaken with his brother, Dr. E. C. Moore, of Detroit, and subsequently with a practitioner in this city. He graduated at the Ohio College of Dental Surgery. He became a member of several societies, among them this, serving as its president only a year ago. He was an honest, upright, Christian gentleman, and his untimely taking off is a genuine loss to the society and the profession of this city.

With one accord we extend to the widow and family our sincerest sympathy in their bereavement, and as long as the association exists his name shall have a place in the list of its honored dead.

(Signed) J. TAFT, E. G. BETTY, A. F. EMMINGER } Committee.

The Committee on Membership proposed the following names: Dr. W. H. H. Hunter, of Cincinnati, O.; Dr. G. S. Junkerman, of Cincinnati, O.; Dr. J. C. Morten, of Brookville, Ind.; Dr. T. J. Phillips, of Canton, O.; Dr. W. P. Lindsey, of Cincinnati, O.

Upon motion, they were elected to membership by cast of the secretary's ballot.

Upon motion, the resolution appointing a committee to attend a preliminary meeting in reference to the International Dental Congress was reconsidered and rescinded.

On motion, a vote of thanks was extended to the retiring officers of the association for their faithful services.

The annual election of officers resulted as follows :

President, Dr. M. H. Fletcher, Cincinnati, O.; First Vice-President, Dr. L. E. Custer, Dayton, O.; Second Vice-President, Dr. Otto Arnold, Columbus, O.; Corresponding Secretary, Dr. H. C. Matlack, Covington, Ky.; Recording Secretary, Dr. H. T. Smith, Cincinnati; Treasurer, Dr. F. A. Hunter, Cincinnati.

PUBLICATION COMMITTEE.

Dr. F. A. Hunter, Cincinnati; Dr. F. W. Sage, Cincinnati, O.; Dr. H. T. Smith, Cincinnati.

COMMITTEE ON MEMBERSHIP.

Dr. O. N. Heise, Cincinnati, O.; Dr. A. G. Rose, Cincinnati, O.; Dr. C. B. Gray, Cincinnati.

COMMITTEE ON ETHICS.

Dr. H. A. Smith, Cincinnati; Dr. J. S. Cassidy, Covington, Ky.; Dr. J. Taft, Cincinnati.

EXECUTIVE COMMITTEE.

Dr. J. R. Callahan, Cincinnati, O.; Dr. E. G. Betty, Cincinnati, O.; Dr. H. C. Matlack, Covington, Ky.; Dr. D. R. Jennings, Cleveland, O.; Dr. C. H. Harroun, Toledo, O.; Dr. E. C. Moore, Detroit, Mich.; Dr. D. G. French, Pittsburg, Pa.; Dr. H. H. Harrison, Wheeling, W. Va.; Dr. H. J. McKellops, St. Louis, Mo.; Dr. M. Stout, Chicago, Ill.; Dr. Hacker, Indianapolis, Ind.; Dr. E. C. Dunn, Louisville, Ky.; Dr. W. H. Morgan, Nashville, Tenn.; Dr. Otto Arnold, Columbus, O.

Adjourned: H. T. Smith, Secretary.

---

Some four hundred of the physicians of Brooklyn have formed a protective association for the purpose of avoiding bad debts, and have published for their own information a black-list of persons who can, but do not, pay their doctor's bills.

